# The dawn of synthetic genomics

**DOI:** 10.1111/j.1462-2920.2008.01581.x

**Published:** 2008-04-01

**Authors:** Michael Y Galperin

**Affiliations:** National Center for Biotechnology Information, National Library of Medicine, National Institutes of Health Bethesda, MD 20894, USA

The first month of 2008 was unusually quiet in terms of microbial genome sequencing. Still, even the relatively short list of newly released genomes includes several interesting environmental microorganisms, such as the anoxygenic phototroph *Chloroflexus aurantiacus*, the toxic bloom-forming cyanobacterium *Microcystis aeruginosa*, and the methylotroph *Methylobacterium extorquens* (Table 1). However, arguably the biggest news was the announcement by J. Craig Venter and colleagues that they ‘have synthesized a 582 970 bp *Mycoplasma genitalium* genome’ (Gibson *et al*., 2008). The authors used chemically synthesized oligonucleotides ∼50 nucleotides in length to assemble ‘cassettes’ 5–7 kb in length, then to join them by *in vitro* recombination to produce intermediate assemblies, gradually increasing in size. Finally, four 144 kb pieces were cloned in *Escherichia coli* as bacterial artificial chromosomes, transferred into yeast and assembled into a full-length genome. The genome of the resulting strain, named *M. genitalium* JCVI-1.0, was virtually identical to the genome of the original strain *M. genitalium* G37. It was not immediately clear whether this technically very challenging and truly monumental work had any purpose beyond just serving as a proof of principle. However, J. C. Venter and colleagues have a record of overcoming enormous technical challenges and launching entirely new areas of biotechnology. It might be simply too early right now to ask them to explain the future of this work. In any case, the era of synthetic biology has officially begun and who knows what kind of molecules people will be synthesizing 10 or 20 years from now.

**Table 1:** Recently completed microbial genomes (December 2007–January 2008).

Species name	Taxonomy	GenBank accession	Genome size (bp)	Proteins (total)	Sequencing centre^a^	Reference
New organisms						
*Renibacterium salmoninarum*	*Actinobacteria*	CP000910	3 155 250	3507	NOAA Fisheries	Unpublished
*Chloroflexus aurantiacus*	*Chloroflexi*	CP000909	5 258 541	3853	JGI	Unpublished
*Microcystis aeruginosa*	*Cyanobacteria*	AP009552	5 842 795	6312	Kazusa	Kaneko *et al.* (2007)
*Bacillus weihenstephanensis*	*Firmicutes*	CP000903-CP000907	5 872 743 (total)	5653	JGI	Unpublished
*Gluconacetobacter diazotrophicus*	*α-Proteo bacteria*	AM889285, AM889287, AM889286	3 944 163 38 818 16 610	3855	RioGene consortium	Unpublished
*Methylobacterium extorquens*	*α-Proteo bacteria*	CP000908	5 471 154	4829	JGI	Unpublished
*Bordetella petrii*	*β-Proteo bacteria*	AM902716	5 287 950	5031	Bielefeld U.	Unpublished
New strains						
*Chlamydia trachomatis* strain L2/434/Bu	*Chlamydiae*	AM884176	1 038 842	874	Sanger Institute	Thomson *et al.* (2008)
*Chlamydia trachomatis* L2b/UCH-1/proctitis	*Chlamydiae*	AM884177	1 038 869	874	Sanger Institute	Thomson *et al.* (2008)
*Rickettsia rickettsii* str. Iowa	*α-Proteo bacteria*	CP000766	1 268 175	1384	Integrated Genomics	Ellison *et al.* (2008)
*Brucella suis* ATCC 23445	*β-Proteo bacteria*	CP000911, CP000912	1 923 763 1 400 844	3241	VBI	Unpublished
*Neisseria meningitidis* 053442	*β-Proteo bacteria*	CP000381	2 153 416	2020	Bejing Genome Ctr.	Peng *et al*. (2008)
*Actinobacillus pleuropneumoniae* serovar 3 str. JL03	*γ-Proteo bacteria*	CP000687	2 242 062	2036	JCVI	Xu *et al.* (2008)
*Coxiella burnetii* RSA 331	*γ-Proteo bacteria*	CP000890, CP000889	2 016 427 37 317	1975	JCVI – San Diego	Unpublished
*Yersinia pestis* Angola	*γ-Proteo bacteria*	CP000901, CP000902, CP000900	4 504 254 114 570 68 190	4045	JCVI	Unpublished

a Sequencing centre names are abbreviated as follows: Bejing Genome Ctr., State Key Laboratory for Moleclular Virology and Genetic Engineering, Microbial Genome Center of Chinese Ministry of Public Health, Beijing, China; Bielefeld U., Center for Biotechnology, Bielefeld University, Bielefeld, Germany; Integrated Genomics, Integrated Genomics, Inc., Chicago, Illinois, USA; JCVI, J. Craig Venter Institute, Rockville, Maryland, USA; JCVI-San Diego, J. Craig Venter Institute, La Jolla, California, USA; JGI, US Department of Energy Joint Genome Institute, Walnut Creek, California, USA; Kazusa, Kazusa DNA Research Institute, Kisarazu, Chiba, Japan; NOAA Fisheries, Northwest Fisheries Science Center, National Marine Fisheries Service, NOAA, Seattle, Washington, USA; RioGene consortium, Universidade Federal do Rio de Janeiro, Instituto de Bioquimica Medica, Rio de Janeiro, RJ, Brazil; Sanger Institute, The Wellcome Trust Sanger Institute, Wellcome Trust Genome Campus, Hinxton, Cambridge, UK; VBI, Virginia Bioinformatics Institute at Virginia Polytechnic Institute and State University, Blacksburg, Virginia, USA.

Returning to the present-day problems, scientists at the Northwest Fisheries Center have sequenced the genome of *Renibacterium salmoninarum*, the causative agent of bacterial kidney disease (kidney granulomatosis) in salmonid fish. *Renibacterium salmoninarum* is a moderately psychrophilic actinobacterium that grows optimally at 15–18°C (Sanders and Fryer, 1980; Fryer and Sanders, 1981). It can survive for several days in fresh or sea water before infecting new fish. In addition, *R. salmoninarum* populates ovarian liquid of infected female fish and can be vertically transmitted to their eggs. The disease was first detected in 1930s in salmon from the river Dee and was initially referred to as ‘Dee disease’. It took more than 20 years to cultivate the organism and recognize its similarity to corynebacteria. The disease still remains poorly understood owing largely to the extremely slow growth of *R. salmoninarum* in pure culture (Hirvelä-Koski, 2008). The genome data are expected to help identify *R. salmoninarum* virulence factors, vaccine candidates and design improved diagnostic tests.

*Chloroflexus aurantiacus* is a facultatively aerobic phototrophic gliding filamentous bacterium, first isolated from the Hakone hot spring area west of Tokyo, Japan (Pierson and Castenholz, 1974). It is the best-studied representative of the phylum *Chloroflexi*, also referred to as *Green non-sulfur bacteria*, and a popular model organism for studying anoxygenic photosynthesis and autotrophic CO_2_ assimilation pathways (Stackebrandt *et al*., 1996; Herter *et al*., 2001). *Chloroflexi* are an early branching bacterial phylum that has retained certain unique properties, including very unusual cell walls (Meissner *et al*., 1988). These organisms are particularly important for understanding the evolution of photosynthesis. In contrast to anoxygenic phototrophs that belong to green sulfur bacteria (*Chlorobi*), *C. aurantiacus* encodes photosynthetic reaction centre of type II, similar to the Photosystem II found in cyanobacteria and green plants, as well as in phototrophic proteobacteria. Accordingly, *Chloroflexi* have been proposed to be the original phototrophs (Oyaizu *et al*., 1987). An alternative, less exciting variant simply implied that *Chloroflexi* have acquired their photosynthetic machinery via lateral gene transfer from ancestral cyanobacteria (Mulkidjanian *et al*., 2006). The sequencing of *C. aurantiacus* genome has had a long history. The first version, consisting of 1142 contigs, was deposited in GenBank back in 2002. In 2005, the number of contigs was reduced to 77, and remained at that stage for more than 2 years. Meanwhile, complete genomes of six members of *Chloroflexi* (three *Dehalococcoides* sp., two *Roseiflexus* sp., and *Herpetosiphon aurantiacus*) had been completed and released to the public. Still the release of the complete genome of *C. aurantiacus* is an important milestone, which will allow many new uses of this model organism.

*Microcystis aeruginosa* is a freshwater planktonic unicellular cyanobacterium with small coccoid cells that form gas vesicles. It is widespread in lakes and ponds around the world and is a common cause of toxic water blooms (Otsuka *et al*., 2001). Its toxicity is due to the production of two major types of toxins, microcystins and cyanopeptolins. Both are cyclic peptides that contain unusual amino acid residues. Microcystin (see PubChem http://pubchem.ncbi.nlm.nih.gov/summary/summary.cgi?cid=445434 or ChEBI http://www.ebi.ac.uk/chebi/searchId.do?chebiId=CHEBI:6925 web sites for representative formulas) is an inhibitor of cellular protein serine phosphatases. It is hepatotoxic and can kill fish, birds and small animals. Cyanopeptolin, also referred to as micropeptin or microcystilide, is a heptapeptide that contains a six-amino-acid-membered ring with a lactone structure between the C-terminus and the hydroxy group of threonine. It is a strong inhibitor of chymotrypsin and related proteases. The genome sequence revealed three non-ribosomal peptide synthetase gene clusters (Kaneko *et al*., 2007). Two of them were responsible for the synthesis of microsystin and cyanopeptolin respectively. The third non-ribosomal peptide synthetase gene cluster and a putative polyketide synthase gene cluster could be involved in production of novel, still unidentified, compounds. Similarly to other cyanobacteria, *M. aeruginosa* carries the full set of photosynthetic genes; however, it encodes a relatively simple two-component signal transduction machinery.

*Bacillus weihenstephanensis* is a Gram-positive, facultatively anaerobic, spore-forming bacterium, a close relative of *Bacillus cereus*. It was recognized as a separate species based primarily on the ability of its strains to grow at low temperatures (< 7°C) and absence of growth above 38°C (Lechner *et al*., 1998). Subsequent multiple-locus sequence typing (MLST) analyses revealed that most psychrophilic *B. cereus*-like isolates belong to *B. weihenstephanensis*, although some psychrophilic strains still had to be assigned to *B. cereus* (Stenfors and Granum, 2001; Sorokin *et al*., 2006). Owing to its tolerance to cold, *B. weihenstephanensis* was reported to be the most common representative of the *B. cereus* group found in frozen soil in Paris area and in Danish sandy loam (Hendriksen *et al*., 2006). Certain strains of *B. weihenstephanensis* have been shown to carry plasmid-encoded genes coding for *B. cereus* haemolysins and enterotoxins. Coupled with the ability of *B. weihenstephanensis* spores to survive heat treatment and give rise to vegetative cells that can grow at refrigeration temperatures, presence of these genes makes *B. weihenstephanensis* a potential food-borne pathogen. Indeed, this organism has been isolated from spoiled whole liquid egg products (Baron *et al*., 2007). The sequenced strain *B. weihenstephanensis* KBAB4 was originally isolated in December 2000 from forest soil at La Minière near Versailles, France, and subsequently identified as *B. weihenstephanensis* using MLST (Sorokin *et al*., 2006). Its genome sequence could shed light on the mechanisms of psychrophilic adaptations in bacilli.

The α-proteobacterium *Gluconacetobacter diazotrophicus* is a nitrogen-fixing member of family *Acetobacteraceae*, originally isolated from sugar cane (Gillis *et al*., 1989), but later found also in association with rice. It is an endophyte that colonizes plant roots, but can propagate to the xylem of the lower stem. *Gluconacetobacter diazotrophicus* can fix N_2_ in the presence of nitrate and is being used as model organism to study the mechanisms and regulation of nitrogen fixation. Sequencing of its genome should help in understanding the physiology of nitrogen-fixing acetic acid bacteria.

Methylotrophic (or, more precisely, methanotrophic) bacteria attract a lot of attention thanks to their ability to utilize natural gas, producing biomass and synthesizing a variety of useful compounds (see Hanson and Hanson, 1996; Trotsenko *et al*., 2005; Hakemian and Rosenzweig, 2007 for reviews). The first methylotroph to undergo genome sequencing was the α-proteobacterium *M. extorquens* strain AM1, a well-characterized model organism. Although its genome sequence has not been finished, it provided a useful insight in the mechanisms of methylotrophy (Chistoserdova *et al*., 2003). The first methylotroph with a complete genome sequence was the γ-proteobacterium *Methylococcus capsulatus* (Ward *et al*., 2004). It was followed by complete genomes of two methylotrophic β-proteobacteria, *Methylobacillus flagellatus* (Chistoserdova *et al*., 2007) and *Methylibium petroleiphilum* (Kane *et al*., 2007). JGI scientists have just released the complete genome of *M. extorquens* strain PA1, which reportedly colonizes plants more efficiently than AM1 strain, and are currently sequencing genomes of four more members of the genus *Methylobacterium*. The availability of complete genomes from three different subdivisions of Proteobacteria opens new possibilities for methylotroph genome analysis and should allow addressing the question of Wood and colleagues (2004) about the causes of obligate methanotrophy. While all sequenced organisms are members of the *Proteobacteria*, methylotrophy has also been found among members of other phyla, such as *Planctomycetes* (Chistoserdova *et al*., 2004). Very recently, papers from three different groups reported isolation of extremely acidophilic methantrophs belonging to the phylum *Verrucomicrobia* (Dunfield *et al*., 2007; Pol *et al*., 2007; Islam *et al*., 2008). These three organisms, isolated from substantially different ecological niches, were all thermophiles capable of growing aerobically at 55–60°C with methane as the sole carbon source. These findings indicate that methanotrophy is far more common in bacteria than previously believed and hold great promise for use of methylotrophs in biotechnology.

All strains of *Bordetella* characterized in the 20th century were animal or avian pathogens, with *Bordetella pertussis* and *Bordetella parapertussis* known as causative agents of whooping cough. In 2001, however, a sample of an anaerobic, trichlorobenzene-dechlorinating consortium taken from sediment of the River Saale near Jena, Germany, was found to contain a *Bordetella*-like organism (von Wintzingerode *et al*., 2001). Unlike other *Bordetella* sp., this isolate, assigned to the new species *Bordetella petrii*, was capable of growing anaerobically by reducing nitrate and/or selenate. Very similar strains were subsequently isolated from soil polluted with chlorinated benzenes (Wang *et al*., 2007), and from patients with mandibular osteomyelitis and chronic suppurative mastoiditis (Fry *et al*., 2005; Stark *et al*., 2007). Thus, *B. petrii* appears to be an opportunistic pathogen, after all, which might prevent its use as a bioremediation agent.

Among the genomes of new strains of previously sequenced species, it is worth noting the detailed description of *Actinobacillus pleuropneumoniae* JL03 genome (Xu *et al*., 2008), published simultaneously with a brief description of the genome of *A. pleuropneumoniae* L20 (Foote *et al*., 2008), which had been released a year earlier. The latter paper appeared in the new ‘Genome Announcement’ section of the *Journal of Bacteriology*. This new section seems to be a very timely initiative, aimed at clearing the backlog of completely sequenced genomes that still remain without proper description or even a suitable citation.
